# Management of Class I and Class II Amalgam Restorations with Localized Defects: Five-Year Results

**DOI:** 10.1155/2013/450260

**Published:** 2013-01-28

**Authors:** Javier Martin, Eduardo Fernandez, Juan Estay, Valeria V. Gordan, Ivar Andreas Mjör, Gustavo Moncada

**Affiliations:** ^1^Restorative Dentistry Department, Dental School, University of Chile, Sergio Roberto Livingstone 943, 8380000 Santiago, Chile; ^2^Department of Restorative Dental Science, Health Science Center, College of Dentistry, University of Florida, Gainesville, FL 32610-0415, USA

## Abstract

Replacement of dental restorations has been the traditional treatment for defective restorations. This five-year prospective clinical trial evaluated amalgam restorations with localized defects that were treated by means of repair or refurbishing. Fifty-two patients (50% female and 50% male, mean age 28.3 ± 18.1 years, range 18–80) with 160 class I and class II defective restorations were included. The study focused on the application of two minimally invasive treatments for localized restoration defects and compared these with no treatment and total replacement as negative and positive controls, respectively. Restorations were assessed by two calibrated examiners according to modified U.S. Public Health Service criteria, including marginal adaptation, anatomic form, secondary caries, and roughness. At five years, recall was examined in 45 patients with 108 restorations (67.5%). The results suggest that repair treatment is as effective as total replacement of restorations with localized defects, reducing biological costs to the patient and providing new tools to the clinician. Refinishing restoration is a useful treatment for localized anatomic form defects.

## 1. Introduction

Amalgam is a restorative material especially suitable for classes I and II restorations in teeth that encounter heavy chewing forces. The advantages of amalgam restorations include resistance to wear, tolerance to a wide range of clinical placement conditions, and excellent load-bearing properties [1–3]. However, amalgam restorations may also present degradation in the intraoral environment due to secondary caries, fracture, marginal breakdown, and wear [4–6].

The traditional solution for those failures has been the complete replacement of the restorations, which may also include minor imperfections in the restorations, and replacement of defective restorations represents a major concern in dental practice, reaching up to 60% of operative dentistry interventions [7]. Consequently, the median survival time (MST) of amalgam varies from 2 to 11 years, but most studies place it at over 5 years [8–10]. 

Complete replacement of restorations has the disadvantages of being time consuming, unnecessary removal of healthy tooth tissue, enlarging preparations and restoration sizes [11, 12], the risk of converting the restoration to an indirect restoration, and the possibility of major injuries in pulp tissues [12–14]. During the last years, new strategies, such as repair and refinishing of localized defects, have shown improvement in the quality of the defective restorations and increased longevity of restorations with minimal intervention [15–17]. Repair rather than replacement of failing restorations is a part of minimally invasive dentistry, preservation of natural tooth structure, early detection of carious lesions, nonsurgical interventions, and a modified surgical approach that includes delayed restoration and smaller tooth preparations with modified cavity designs [18].

The aim of this clinical trial was to assess the long-term performance of two minimally invasive clinical procedures, repair and refinishing, as treatments for localized defects of classes I and II amalgam restorations. 

## 2. Methods and Materials

The Institutional Research and Ethical Board of the Dental School at the University of Chile approved this randomized clinical trial (project PRI-ODO-0207). Only faculty members were allowed to provide the restorative treatment. 

### 2.1. Study Design

Fifty-two patients, 26 female (50%) and 26 male (50%), were recruited at the Operative Dentistry Clinic at the Dental School, University of Chile, Santiago. Patients were aged 18 through 80 years (mean age, 28.3 ± 18.1 years). A total of 160 defective restorations (97 class I, 63 class II) presenting one or more clinical features that deviated from ideal were included in the study. The restorations' clinical conditions ranked from Bravo to Charlie according to the United States Public Health Service (USPHS)/Ryge criteria (see Table 1). 

#### 2.1.1. Inclusion Criteria

Patients with localized deficiencies of amalgam restorations that were clinically judged to be suitable for repair or refinishing according to USPHS criteria (Table 1), patients with more than 20 teeth, restorations in functional occlusion with an opposing natural tooth and at least one proximal contact area with an adjacent tooth, patients older than 18 years old, and patients who signed the consent form and completed a registration form were included in the study. 

#### 2.1.2. Exclusion Criteria

Patients with contraindications for regular dental treatment based on their medical history, patients who had special aesthetic requirements that could not be solved by the alternative treatments, patients with xerostomia or who were taking medication that significantly decreased salivary flow, patients with high caries risk, and patients with psychiatric or physical diseases that interfered with tooth hygiene were excluded from the study. 

#### 2.1.3. Treatment Group Criteria

One hundred sixty defective restorations were evaluated in accordance with USPHS criteria and assigned according to the following criteria.

1. Restorations with clinically diagnosed secondary caries (Charlie) or undercontoured anatomical form defects (Bravo) were randomly assigned to the repair or replacement group (randomization was performed by Power Analysis and Sample Size System in PASS software v. 2008, Keysville, UT, USA). Diagnosis of active secondary caries was made according to Ekstrand's criteria [17].
2. Restorations with overcontoured anatomic form, luster, or roughness defects were randomly assigned to the refinishing (Bravo and Charlie) or no-treatment group (Bravo).
3. Restorations with marginal defects (Bravo) were randomly assigned to either the repair, refinishing, replacement, or no-treatment group.

The treatment groups were labeled (A): repair (*n* = 19); (B): refinishing (*n* = 64); (C): replacement (*n* = 21); (D): no treatment (*n* = 56).

### 2.2. Restoration Assessment

The quality of the restorations was scored according to modified USPHS/Ryge criteria [19]. Two examiners underwent calibration exercises (JM and EF, Cohen's Kappa interexaminer coefficient 0.74 at baseline and 0.87 at five years). The examiners assessed the restorations independently by direct visual and tactile examination (mouth mirror number 5, Hu Friedy Mfg. Co. Inc., Chicago, IL, USA, and explorer number 23 Hu Friedy) and indirectly by radiographic examination (Bite Wing) at baseline (immediately after treatment) and each year up to five years after treatment. The four examined parameters were marginal adaptation (MA), anatomic form (A), surface roughness (R), and secondary caries (SC) (Table 1). If any difference was recorded between the two examiners, and if they did not reach an agreement, a third clinician who also underwent calibration exercises (GM) made the final decision. 

A change from Bravo to Alpha was considered an improvement, and a change from Alpha to Bravo represented deterioration. 

### 2.3. Treatment Groups

- Repair: carbide burs were used to explore the defective margins of the restorations, beginning with the removal of part of the amalgam restorative material adjacent to the defect. Once this material was removed and the exploratory cavity preparation did not include any stained or soft tooth tissues, a dispersed-phase amalgam (Original D, Wykle Research, Inc, Carson City, NV, USA) was used to repair the preparation. Mechanical retention was employed inside the existing restoration. Rubber dam isolation was used for this procedure.
- Refinishing: defective areas of the amalgam restoration were smoothed using carbide burs (numbers 12 and 30, Brasseler USA, Savannah, GA, USA). On the occlusal and buccal/lingual surfaces, silicone-impregnated points (Brownie/Greenie/Supergreenie, Shofu Dental Corporation, Menlo Park, CA, USA) were used for polishing. When the proximal area was affected, the defective areas were smoothed with aluminum oxide finishing strips (Sof-Lex, 3M ESPE).
- Replacement: the defective restoration was totally removed and replaced with a new amalgam (Tytin, Kerr Corporation, Orange, CA, USA). Rubber dam isolation was used for this procedure.
- No treatment: the defective amalgam restorations did not receive any treatment.

Patients were recalled each year for up to five years for clinical evaluation by the same blinded examiners, applying the same criteria used at baseline. Failed restorations were removed from the study and treated according to their diagnosed needs.

### 2.4. Statistical Analysis

The results of each group in terms of degradation were analyzed by the Wilcoxon nonparametric test to compare the pre- and postoperative conditions. Additionally, the performance of all groups was contrasted using the *Z* test to determine the differences between upgrade and downgrade restoration quality. The MST of the restorations was determined by the Kaplan-Meier test at each annual recall examination. The statistical significance was set at 95% or *α* = 0.05. SPSS15.0 (SPSS Inc, Chicago, IL) was used for statistical analysis. 

## 3. Results

From the original cohort of 52 patients with 160 restorations, 45 patients with 108 restorations were assessed (67.5%) at the fifth year (66 class I and 42 class II restorations). The distribution of the restorations in the groups was as follows: refinishing (*n* = 46), repair (*n* = 11), replacement (*n* = 15), and no treatment (*n* = 36). 

During the five-year followup, 52 restorations (32.5%) were lost to follow up due to orthodontic treatment where restorations were covered by metallic bands (*n* = 3), address changes or no attendance (*n* = 39), and restorations that presented a Charlie rating during a prior study observation (*n* = 10). The latter were fully replaced and removed from the study.

The results are presented as percentages of Alpha ratings in the different groups. After an initial improvement in all treatment groups, all groups showed a trend to downgrade during the observation period in all parameters, except in secondary caries. Throughout the observation period, a Charlie rating was observed in only a small number of restorations (10/160, 6.25%): 3 at the first year, 2 at the second, 2 at the third, 1 at the fourth, and 2 at the fifth.

### 3.1. Marginal Adaptation

**Figure 1:**
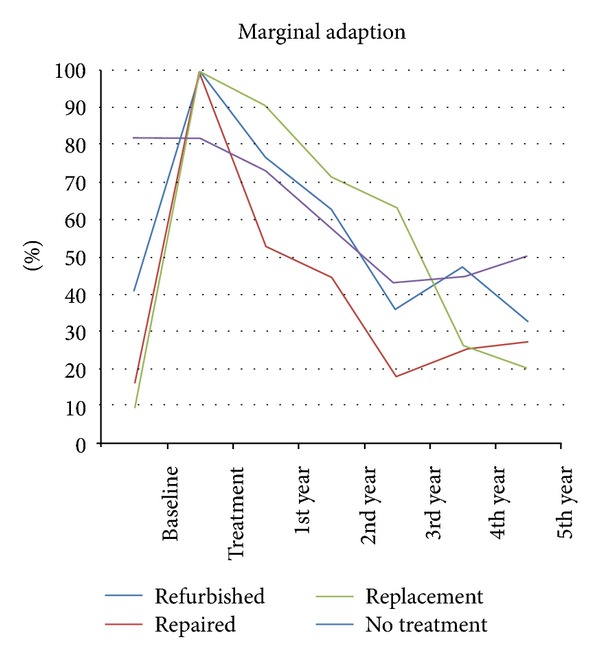
Marginal adaptation curve of all groups, separated by year, expressed as Alfa-rated restorations.

The refinishing and repair groups presented no difference in Alpha-rated restorations between the baseline and the fifth-year examination regarding marginal adaptation. In contrast, the replacement group presented more Alpha-rated restorations at five years than at baseline (*P* = 0.021). No-treatment group showed a reduction of Alpha-rated restorations between the baseline and five-year evaluations (*P* = 0.029) (Figure 1).

The Kaplan-Meier test showed that the refinished and no-treatment groups showed a median survival time (MST) of three years for marginal adaptation after five years. The repair and replacement groups each showed a MST of four years (Figure 8).

### 3.2. Anatomic Form

**Figure 2:**
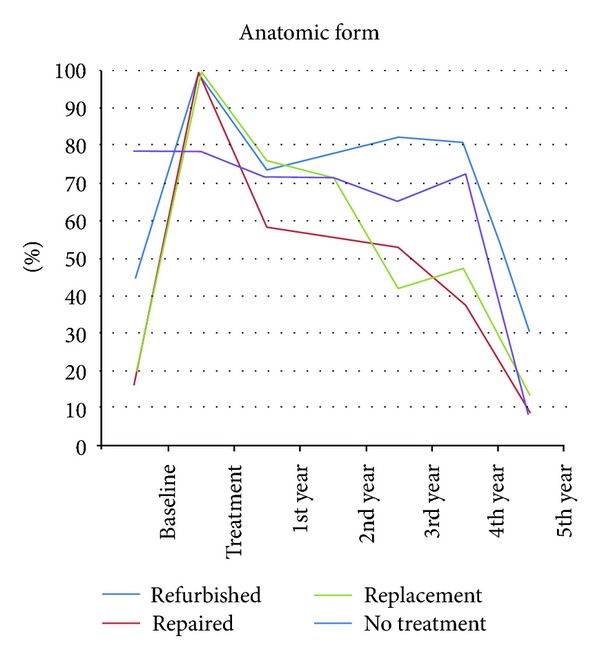
Anatomic form curve of all groups separated by year, expressed as Alfa-rated restorations.

Regarding the anatomic form parameter, the three treated groups showed no difference between baseline and five years. No treated group showed a downgrade in Alpha-rated restorations (Figure 2). Compared to the treated groups, the no-treatment group showed significant downgrade. All groups showed a MST of five years (Figure 7).

### 3.3. Surface Roughness

**Figure 3:**
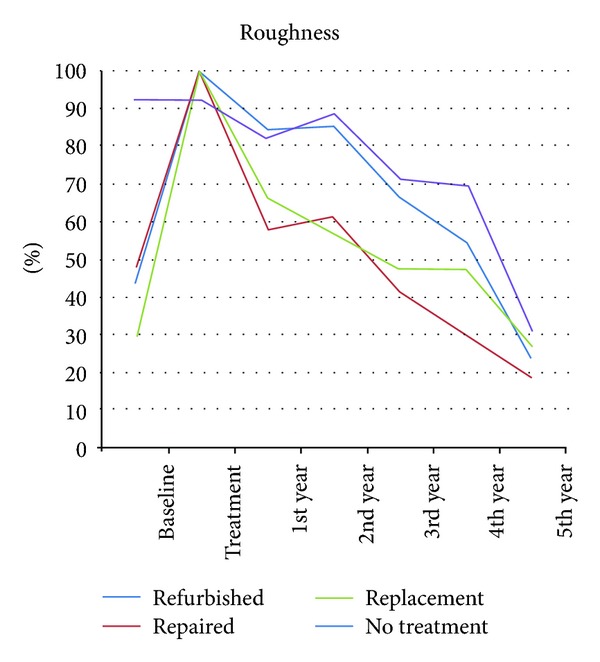
Roughness curve of all groups separated by year, expressed as Alfa-rated restorations.

All treatment groups maintained the same clinical condition as presented at baseline (*P* > 0.05). No treated group showed a significant downgrade in surface roughness after five years (*P*⩾0.005) (Figure 3). The refinishing, replacement and no-treatment groups showed a MST of five years, and the repair group showed a MST of four years (Figure 5).

### 3.4. Secondary Caries

**Figure 4:**
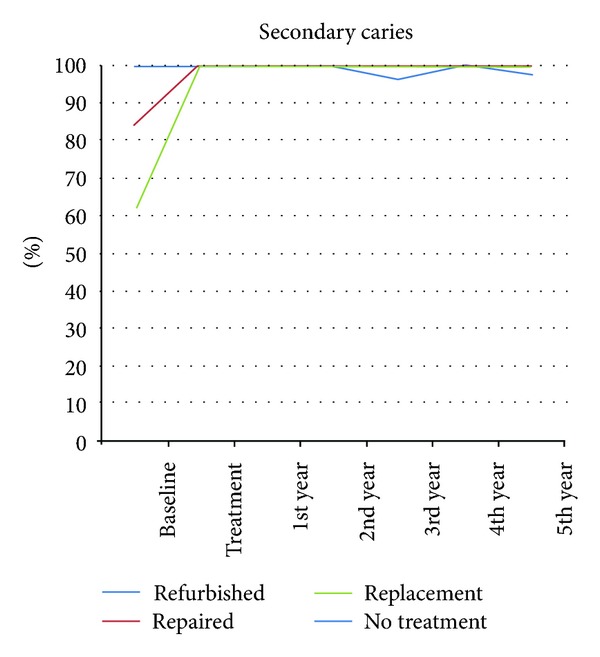
Secondary caries curve of all groups separated by year, expressed as Alfa-rated restorations.

**Figure 5:**
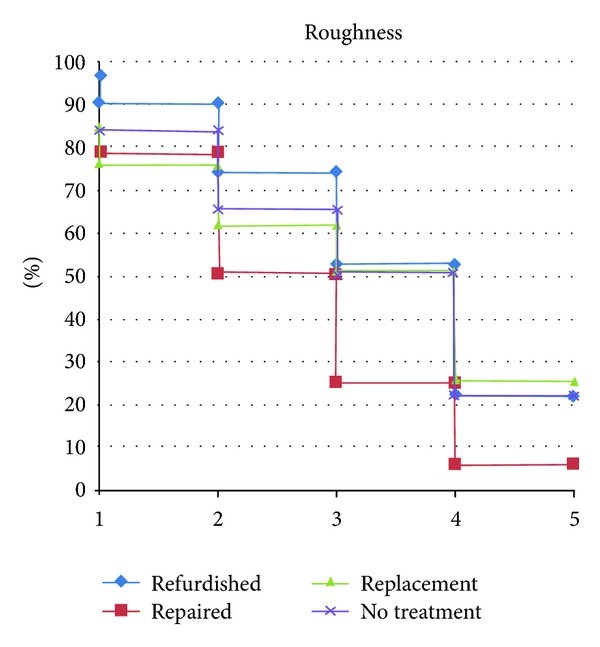
Median survival time of roughness separated by groups after five years by Kaplan Meiers test.

**Figure 6:**
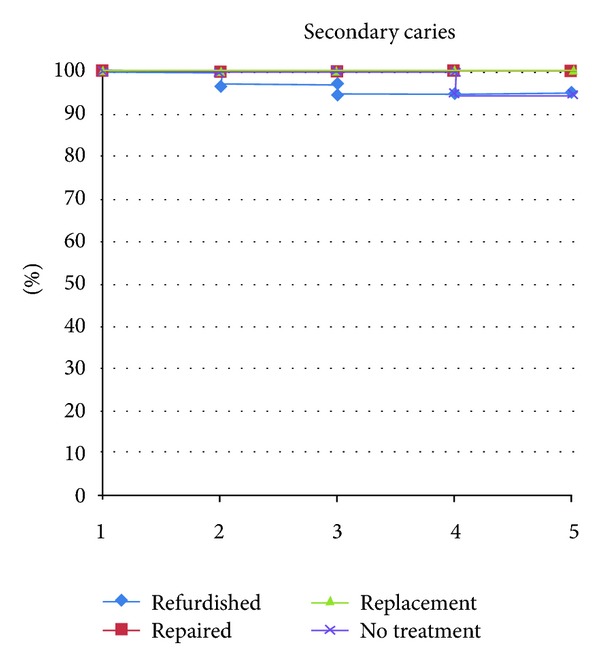
MST of secondary caries separated by groups.

**Figure 7:**
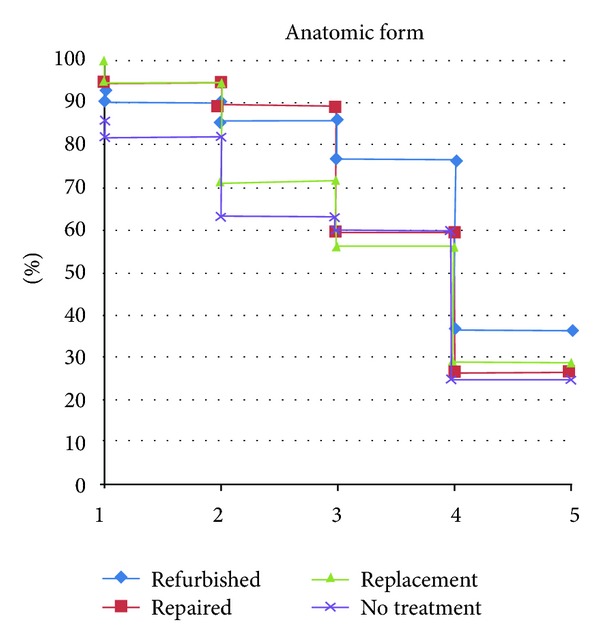
MST of anatomic form separated by groups.

**Figure 8:**
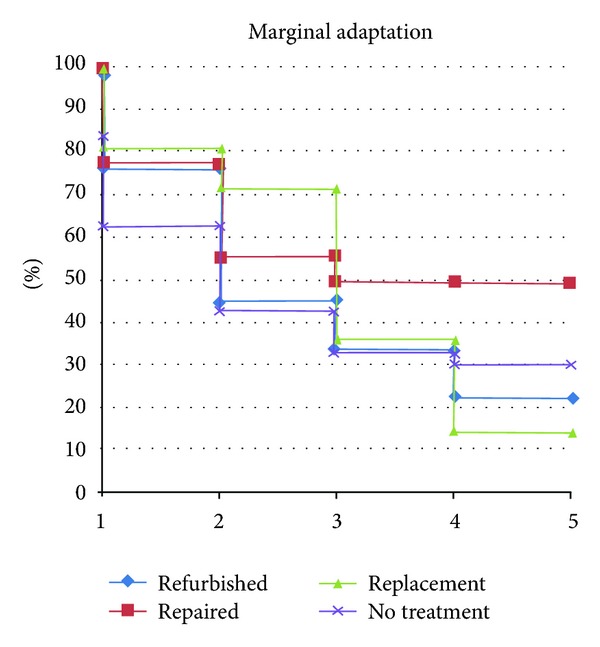
MST of marginal adaptation separated by groups.

The repair group showed a nonsignificant improvement in secondary caries after five years (*P* = 0.157), while the replacement group had a significant improvement (*P* = 0.046). The refinishing (*n* = 1), and no-treatment groups (*n* = 2) presented a low rate of caries lesions (Figure 4). All groups showed a MST of at least five years (Figure 6).

## 4. Discussion

Amalgam longevity is an important issue for patients, governments, and dentists to define the cost of dental treatment. Minimal-intervention dentistry, such as repair or refinishing of localized defects of restorations, could increase the longevity of the amalgam restorations and reduce patient stress regarding treatment cost. Repair and refinishing showed a high level of clinical acceptance by patients in this study. Most of the restorations' performance was assessed as clinically acceptable, including Alpha or Bravo ratings in all experimental groups. Only 6.3% were evaluated as Charlie during the five-year observation period. The success of repair and refinishing allowed a significant increase in the lifetime of the original restorations with minimal intervention, as most of these procedures could be performed without dental anesthesia. 

In general, the results show that repairing and refinishing restorations with localized defects are effective and increase the MST of the restorations. This study showed an association between the type of treatment and prognosis, assuming clinical criteria for restoration repair instead of traditional replacement based on quality assessment and the MST of those procedures. The choice of refinishing or repair resulted in tooth tissue preservation instead of unnecessary tooth structure removal, as in the case of the replacement group [17, 19, 20]. The present study did not show a biological risk for the teeth: there were no tooth fractures, a low rate of restoration failures, and no pulp injuries. These results are explained by the use of noninvasive techniques.

The main reason for restoration failures is secondary caries lesions located at the margins of the restorations. These lesions should be clinically differentiated from stained and ditched margins in order to find soft dental tissue or carious areas.

Random assignation in our study was carried out after considering the types of restoration defects. It was not possible to allocate the restorations completely randomly because there are ethical concerns with, for example, secondary caries. Additionally, some localized defects will not improve with minimally invasive treatment, for instance in the case of undercontoured restorations in the refinishing group.

Most of the dentists were traditionally trained in replacement techniques. Only recently have a number of dental schools included restoration repair in their educational programs, which could explain why repair is not popular yet in operative dentistry [21]. 

### 4.1. Repair

**Figure 9:**
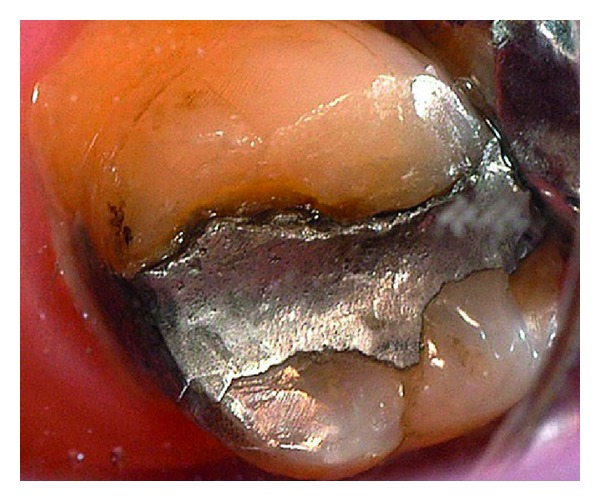
Baseline of a defective amalgam margin of the palatal cusp of a second upper molar. The restoration had served for 17 years. A small cavity was cut initially in the amalgam restoration until sound enamel and dentin could be seen at the pulp floor. The preparation was then repaired with amalgam.

**Figure 10:**
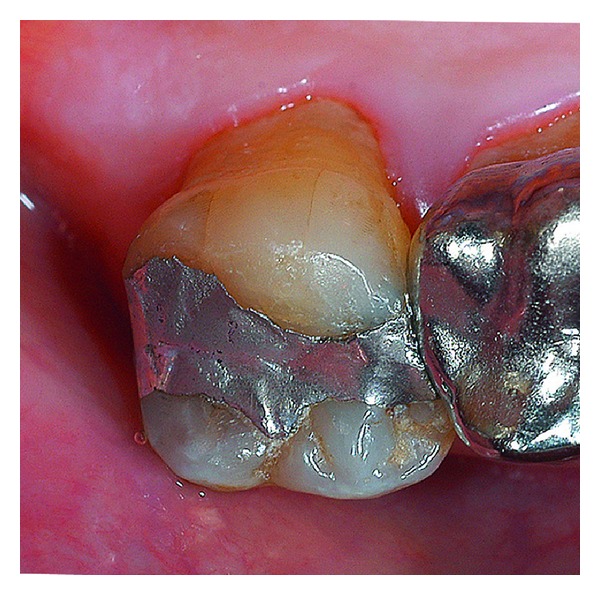
Amalgam restoration immediately after repaired.

**Figure 11:**
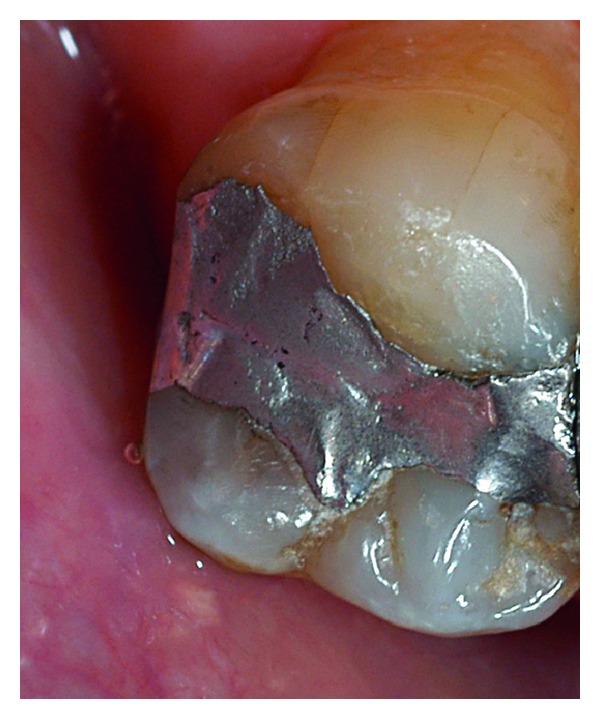
Control 1 year after treatment, where the marginal gap remains filled, the surface of the repaired amalgam presented irregularities, related with patient's occlusion.

**Figure 12:**
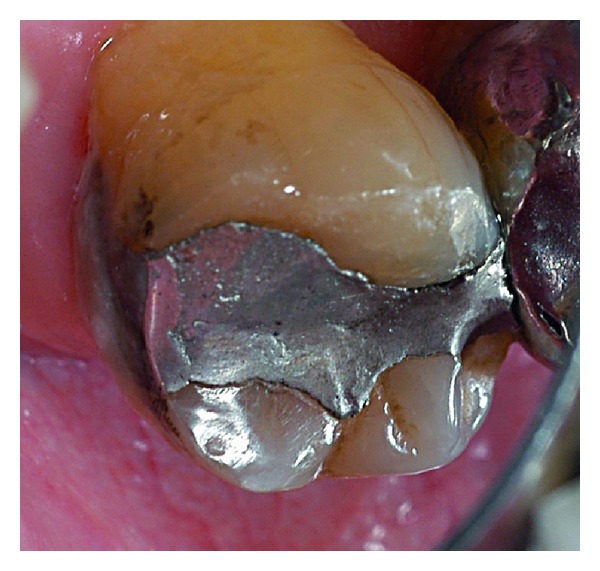
Control 2 years after, showing roughness modifications similar one year control.

**Figure 13:**
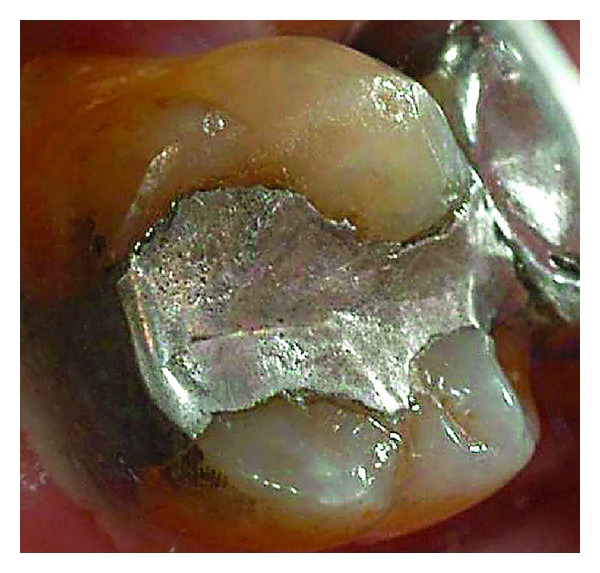
3 years control, with increases of the surface damage including amalgam microfracture, in the disto-palatal edge.

**Figure 14:**
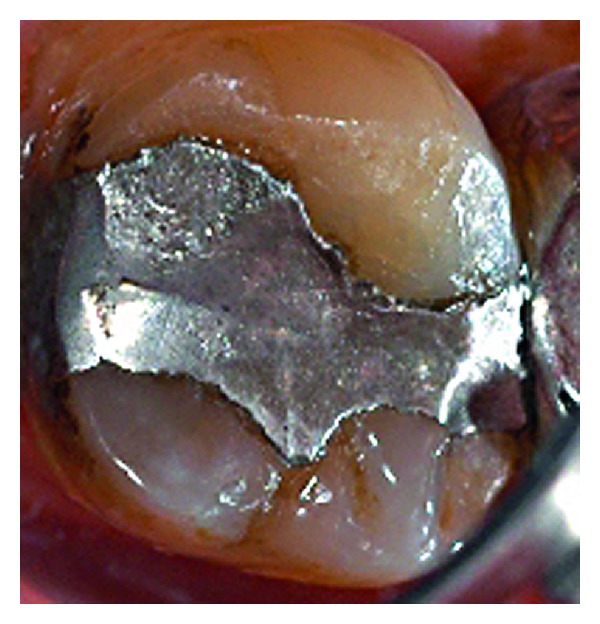
4 years control, showing increases of the surface and marginal damage.

**Figure 15:**
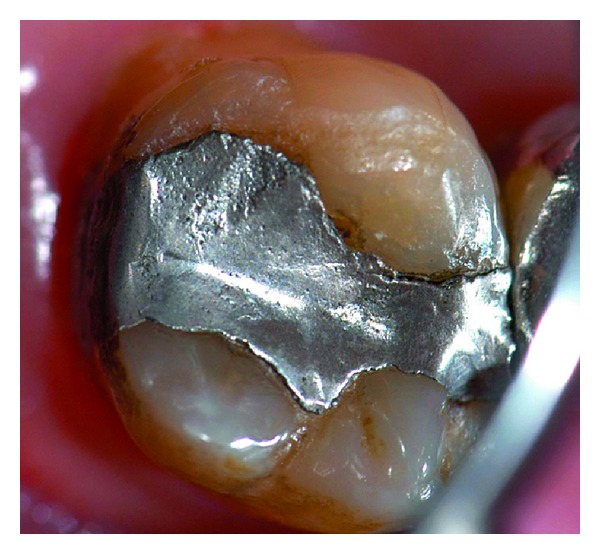
5 years control, showing increases of surface damage, margin fractures of the amalgam and gap appears again, lower than at baseline.

After five years, 96.3% of the restorations presented Alpha ratings for secondary caries, with no significant differences between repair and replacement (see case of repair Figures 9–15). According to this observation, repair must be considered a conservative procedure and can be used safely when there is a small caries lesion with easy access. Thus, this intervention is effective in controlling dental caries lesions. Additionally, no disadvantages were observed regarding repairing restorations with secondary caries. This finding is consistent with previous research, which indicated that the presence of secondary caries is a localized process originating from the surface and not involving the entire restoration [22–25].

Fifty percent of the repaired restorations had an Alpha rating after 2 years in marginal adaptation. This might be explained by the fact that other clinical conditions, such as cavity design, occlusal contacts, and bruxism, were not modified. If marginal adaptations fail, it is possible to reduce marginal discrepancies by applying other minimally invasive procedures, such as marginal flowable resins or marginal sealant, which is a practical, easy, and fast alternative to sealing the gap with pits and fissure sealant. Marginal sealants perform better than repair over time [11, 17, 19, 20]. 

Full replacement of restorations promotes less preservation of healthy tooth tissues and is also more time consuming than restoration repair, and yet it is the most prevalent procedure in general dental practice [7, 11–14, 26]. A recent study suggests that repaired restorations could outlast restorations that have been replaced, and one possible reason for this is that most of the original restoration is kept intact [19]. Although the use of resin-based composite to repair amalgam restorations is considered an appropriate process whenever a proper surface conditioned technique is applied [27, 28], restorations were repaired with amalgam based on the low cost and long-term effectiveness of this material [29]. 

### 4.2. Refinishing

Fifty percent of the restorations maintained Alpha-rated anatomic form and surface roughness for at least 4 years in restorations that were refinished. These two parameters were the ones that suffered the greatest deterioration over time: 30.4% and 23.9% of restorations were Alpha-rated for these two parameters after 5 years, compared to 8.3% and 30.6%, respectively, in the control group (*P* < 0.001). 

Refinishing could be considered a preventive measure because it reduces the possibility of plaque accumulation, as the restoration may achieve an anatomical form similar to the tooth, making it favorable for improvements towards restoration function and longevity [19, 30–32].

Prior to the refinishing procedure, radiographic examination is mandatory to establish the thickness of the restorations because in shallow restorations the dentin could be exposed or the mechanical properties of the restoration could be impaired. This problem could be avoided by analyzing bite-wing radiographs in the same way that caries lesions are detected. In general, refurbishing is recommended only for improving contoured defects.

### 4.3. Replacement

Full replacement of the restoration did not present secondary caries during the study period, similar to repair. In general, replacement showed the same trend of downgrade as observed in other groups, but it had an increased Alpha rating for marginal adaptation (greater than the refinishing and no-treatment groups), and it had a similar performance to the repair group. Regarding secondary caries and surface roughness, replacement presented the same performance as the other groups.

### 4.4. Control Group

The most relevant downgrade was observed in the no-treatment group regarding anatomic form. This finding support the idea that it is necessary to treat small localized defects of amalgam restorations in order to prevent future damage.

Marginal adaptation was the only parameter that was treated in all four groups. For this reason, it could be considered the only parameter for which it is possible to compare the performance of the four treatments. In this context, the replacement group presented the best performance, as it was the only one that showed more Alpha-rated restorations at five years than at baseline. The no-treatment group showed a significant downgrade in the period of the study.

## 5. Conclusions

The present five-year clinical study supports the concept that repair treatment is as effective as total replacement of restorations with localized defects and reduces biological costs to the patient. Refinishing is useful for treating localized anatomic form defects in existing amalgam restorations.

## 6. Clinical Relevance

Minimally invasive treatments of defective amalgam restorations presented similar results to the restorations that were replaced.

## Figures and Tables

**Table 1:** U.S. Public Health Service/Ryge clinical criteria.

Clinical characteristic	Alfa	Bravo	Charlie
Marginal adaptation	Explorer does not catch when drawn across the restoration/tooth interface	Explorer falls into crevice when drawn across the restoration/tooth interface	Dentin or base is exposed along the margin

Anatomic form	The general contour of the restorations follows the contour of the tooth	The general contour of the restoration does not follow the contour of the tooth	The restoration has an overhang

Surface roughness	The surface of the restoration has not any surface defects	The surface of the restoration has minimal surface defects	The surface of the restoration has severe surface defects

Secondary caries	There is no clinical diagnosis of caries	NA	There is clinical diagnosis of caries
